# Investigation of Antioxidant Mechanisms of Novel Peptides Derived from Asian Swamp Eel Hydrolysate in Chemical Systems and AAPH-Induced Human Erythrocytes

**DOI:** 10.3390/antiox13080888

**Published:** 2024-07-23

**Authors:** Xiao Wang, Bingjie Chen, Khushwant S. Bhullar, Hang Yang, Xiaohu Luo, Juan Fu, Hongru Liu, Di Su, Dapeng Sun, Yongjin Qiao, Wenzong Zhou

**Affiliations:** 1 Crop Breeding and Cultivation Research Institution, Research Center for Agricultural Products Preservation and Processing, Shanghai Academy of Agricultural Sciences, Shanghai 201403, China; zhenhans2005@gmail.com (X.W.); chenbingjie0204@126.com (B.C.); maryfly@live.cn (J.F.); 20200203@saas.sh.cn (H.L.); sundapeng@saas.sh.cn (D.S.); 2 Department of Agricultural Food & Nutritional Science, University of Alberta, Edmonton, AB T6G 2P5, Canada; bhullar@ualberta.ca; 3 Key Laboratory of Integrated Rice-Fish Farming Ecosystem, Ministry of Agriculture and Rural Affairs, Shanghai Academy of Agricultural Sciences, Shanghai 201403, China; yanghangqu@saas.sh.cn; 4 Zhejiang-Malaysia Joint Research Laboratory for Agricultural Product Processing and Nutrition, College of Food Science and Engineering, Ningbo University, Ningbo 315832, China; xh06326@gmail.com; 5 School of Pharmacy, Shanghai Jiao Tong University, 800 Dongchuan Road, Shanghai 200240, China; sudi@sjtu.edu.cn

**Keywords:** antioxidant peptides, Asian swamp eel, molecular docking, erythrocyte morphoprotection, non-enzymatic and enzymatic antioxidant systems

## Abstract

Sixteen novel antioxidant peptides from Asian swamp eel (ASE) were identified in previous studies. However, their chemical and cellular antioxidant mechanisms remain unclear. Molecular docking of these peptides with ABTS and DPPH radicals revealed the critical role of hydrogen bonding and Pi–Pi stacking hydrophobic interactions between hydrophobic amino acid residues and free radicals. Residues, such as tryptophan, proline, leucine, and valine, played significant roles in these interactions. All these peptides exhibited notable erythrocyte morphoprotective effects in a model of AAPH-induced oxidative damage of human erythrocytes. Erythrocyte hemolysis was reduced primarily through the modulation of both non-enzymatic (GSH/GSSG) and enzymatic antioxidant systems (SOD, CAT, and GSH-Px) by these peptides. A decrease in levels of MDA, LDH release, and hemoglobin oxidation was observed. Among the peptides, VLYPW demonstrated superior chemical and cellular antioxidant activities, which may be attributed to its higher levels of tyrosine and tryptophan, as well as to its increased hydrophobic amino acid content.

## 1. Introduction

Endogenous or exogenous stimuli induce the body to produce excessive free radicals, which leads to oxidative stress, damages biomolecules (e.g., DNA, proteins, lipids, etc.), and ultimately triggers cellular dysfunction and disease [1,2]. The dynamic balance of free radicals is capable of being maintained by antioxidant defense systems, encompassing non-enzymatic antioxidant systems and antioxidant enzyme systems [3]. Notably, antioxidant peptides can react directly with free radicals or activate the body’s antioxidant defense system to maintain the balance between oxidative damage and antioxidant defense [4,5]. In recent years, considerable attention has been drawn to the preparation and extraction of antioxidant peptides derived from agricultural products, due to their ease of absorption, high activity, safety, and bioavailability [6,7]. Asian swamp eel (*Monopterus albus*), recognized as a high-protein and low-fat fish, possesses a rich variety of amino acids and has high nutritional value, rendering it a high-quality raw material for the preparation of natural antioxidant peptides [8].

Chemical-based antioxidant assays are widely utilized due to their simplicity, speed, and sensitivity, which enables the rapid evaluation of antioxidant activity. In our previous study, sixteen novel antioxidant peptides (AVLW, VPWP, VWPS, WGWP, DHPWH, LLGPW, VLYPW, VYGPW, WGGPL, WPDAR, WPYVT, LLVYPW, WDGTGR, PSWVPPA, WGDLSPK, and HWDGSLPR) from ASE hydrolysate were identified and screened. In vitro antioxidant studies demonstrated that all fifteen peptides, with the exception of VWPS, exhibited greater antioxidant potency than the standard antioxidant, Trolox. A search of the NCBI database unveiled AVLW, VWPS, VPWP, and HWDGSLPR as new peptide sequences [9,10,11]. The active sites of these sixteen peptides were predicted using quantum chemical calculations and confirmed through methylation of the active sites [12]. However, the mechanism underlying the interaction of these active peptides with free radicals remains unclear. It has been reported that antioxidant peptides possess the capability to interact with free radicals, thereby either scavenging or neutralizing them [3]. The utilization of molecular docking technology to probe into the structure–activity relationships, interaction sites, and mechanisms between peptides and free radicals holds promising potential in the realms of research, design, and synthesis of antioxidant compounds [13,14,15]. Through molecular docking, it was determined by Fan et al. that the antioxidant capacity of MYGAVTPVK, NWEKIR, APGIIPR, and RWWQLR was mainly realized through the formation of hydrogen bonds between their amino acid residues and free radicals [16]. A study conducted by Wen et al. revealed that good antioxidant activity was observed in the antioxidant peptide of watermelon seed, RDPEER [3]. Two hydrogen bonds with DPPH radicals were formed by Arg-1 and Arg-6 residues, and one hydrogen bond, along with one hydrophobic interaction with DPPH radicals, was formed by Glu-5 residues; two hydrogen bonds with ABTS radicals were formed by Arg-1 and Glu-5 residues; and three hydrophobic interactions with ABTS radicals were formed by Asp-2, Glu-4, and Arg-6 residues. A study carried out by Agrawal et al. demonstrated that the serine and threonine residues in the millet antioxidant peptides, TSSSLNMAVRGGLTR and STVGLGISMRSASVR, were effective in forming hydrogen bonds with DPPH radicals and ABTS radicals, and exerted antioxidant effects [17].

In vitro chemistry and computer simulation methods are found to be deficient in considering physiological conditions, bioavailability, and metabolism [18]. Therefore, a comprehensive assessment of antioxidant activity often requires integration with cellular evaluation [19]. Commonly utilized cell lines include Caco-2, HepG2, HeLa, HUVEC, SH-SY5Y, PC12, and RAW264.7 cells [20]. Erythrocytes, characterized by their high content of polyunsaturated fatty acids and redox-active hemoglobin molecules, are particularly prone to oxidative stress [21]. Hence, erythrocytes are regarded as an ideal cellular model for investigating redox biology. Enhancing the antioxidant properties of erythrocytes ameliorates sickle cell anemia [22], improves erythrocyte storage conditions [23,24], and positively affects both erythrocyte phenotype and post-transfusion recovery. The main sources of endogenous erythrocyte oxidants are superoxide radicals and hydrogen peroxide [25]. AAPH is capable of generating free radicals, especially superoxide radicals. Therefore, the AAPH treatment of erythrocytes was used in this thesis to model erythrocyte oxidation [26].

In our previous study, sixteen novel ASE antioxidant peptides were identified, among which four new peptide sequences were included. In this study, the dominant conformations and interactions of the sixteen peptides with free radicals (ABTS and DPPH) were analyzed by molecular docking to reveal their antioxidant mechanisms. To further investigate the cellular antioxidant activities of these sixteen peptides, the protective effects on the morphology of human blood erythrocytes in the AAPH-treated oxidative stress model were evaluated, and the protective mechanisms of the sixteen peptides against the antioxidant defense systems (including the non-enzymatic antioxidant system and antioxidant enzyme system) of human blood erythrocytes were elucidated. The results of this study will contribute to the promotion of peptide resource development in ASE as well as the exploration of potential applications of these antioxidant peptides in functional foods.

## 2. Materials and Methods

### 2.1. Materials

Human blood erythrocytes (1%, *v*/*v*) were purchased from Cenxi Hongquan Biotechnology Co. (Guangzhou, China) 2, 2′-azido[2-methylpropionamide] dihydrochloride (AAPH) was purchased from Beijing Essan Huitong Technology Co. (Beijing, China) Peptides, including AVLW, VPWP, VWPS, WGWP, DHPWH, LLGPW, VLYPW, VYGPW, WGGPL, WPDAR, WPYVT, LLVYPW, WDGTGR, PSWVPPA, WGDLSPK and HWDGSLPR, were purchased from Nanjing Jiepeptide Biotechnology Co., Ltd. (Nanjing, China) The purity of all peptides was higher than 95% after UPLC-MS/MS analysis.

The experimental kits, including BCA protein quantification, catalase (CAT) activity, malondialdehyde (MDA) levels, lactate dehydrogenase (LDH) activity, glutathione peroxidase (GSH-Px) activity, superoxide dismutase (SOD) activity, reduced glutathione (GSH), and oxidized glutathione (GSSG) assays, were purchased from Nanjing Jianjian Haihao Biotechnology Co., Ltd. (Nanjing, China). All other reagents used were of analytical grade and sourced from China.

### 2.2. Molecular Docking of Antioxidant Peptides with Free Radicals (ABTS, DPPH)

Molecular docking analysis was performed to study the interactions of peptides with free radicals. The three-dimensional structures of ligands ABTS (CID:5360881) and DPPH (CID:2735032) were obtained from the PubChem database [16]. Subsequently, the ligand files, in sdf format, were converted to pdb format using OpenBabel 2.3.1 software. These files were then imported into Autodock 4.2.6 software to determine the rigidity of the ligands and configure the torsionable key, and were then saved in pdbqt format for subsequent grid calculations [12,27]. The three-dimensional structure of the antioxidant peptides were drawn with ChemDraw 20.0. Following this, the ligands and radicals were docked using Autodock Tools 1.5.6, and the complexes with the highest scores were considered as the best docking results [28]. Finally, the complexes formed by radicals and peptides were visualized using PyMol 2.3.0 and Discovery Studio 4.5 [29].

### 2.3. Cell Culture and Modeling

An AAPH-induced oxidative damage model in human blood erythrocytes was established following the method of Du et al. [30]. Initially, 1% human blood erythrocytes were centrifuged at 8000 rpm for 10 min at 4 °C to separate them from the plasma. The erythrocyte precipitate was then collected and washed four times with phosphate buffer salt (PBS) buffer (pH 7.4), consisting of 137 mM NaCl, 2.7 mM KCl, 8.1 mM Na_2_HPO_4,_ and 1.5 mM KH_2_PO_4_. After washing, a specific volume of the erythrocyte precipitate was diluted with PBS and thoroughly mixed to create a 10% erythrocyte suspension [5].

Subsequently, 100 μL of the 10% erythrocyte suspension was dispensed into each well of a 96-well culture plate. Then, 50 μL of either the sample solution (sample group) or PBS (control group and AAPH group) was added to each well. The plates were then incubated in a biochemical incubator at 37 °C for 30 min. After pre-incubation, 100 μL of a 200 mM AAPH solution was added to induce oxidative damage, and the plates were further incubated for 3 h. In the control group, the erythrocyte suspension was mixed with the PBS buffer and subjected to the same incubation conditions. Both the samples and AAPH were prepared using the PBS buffer, as previously described [5,31].

### 2.4. Scanning Electron Microscopy (SEM) Observation of Erythrocyte Morphology

The erythrocytes, treated with the different peptides from Section 2.3, were precipitated and then incubated with 2.5% glutaraldehyde for 12 h at 4 °C to immobilize them [26]. Subsequently, the cells were centrifuged at 4000 rpm for 5 min at 4 °C, and the supernatant was carefully discarded. The cells underwent three rinses with PBS to remove excess glutaraldehyde. Following this, a small aliquot of the fixed erythrocyte samples was thinly spread onto coverslips and air-dried at room temperature, followed by gradual dehydration with ethanol concentrations of 30%, 50%, 70%, and 90% [27]. The coverslips, with affixed erythrocyte samples, were then coated with gold using a gold sprayer (ETD-2000, Beijing Aliberton Science and Technology Development Co., Ltd., Beijing, China). Finally, the samples were examined and imaged under an electron microscope (EVO 18, Carl Zeiss, Jena, Germany) [5].

### 2.5. Determination of Hemolysis Rate

The erythrocyte reaction solution from Section 2.3 was transferred to a 2 mL centrifuge tube, followed by the addition of 600 µL of the PBS buffer. After centrifuging the mixture at 8000 rpm for 10 min at 4 °C, 200 µL of the supernatant was carefully collected and transferred to a 96-well transparent microtiter plate. The absorbance, at 540 nm, was measured and recorded as A_PBS_. This procedure was repeated with an equal amount of the 10% erythrocyte suspension. Complete erythrocyte lysis was ensured using ultrapure water at 4 °C, and the absorbance at 540 nm was recorded as A_water_. The hemolysis rate was calculated using the following formula: hemolysis rate (%) = (A_PBS_/A_water_) × 100% [5,30].

### 2.6. Measurement of Hemoglobin Oxidation Rate

The oxidation rate of hemoglobin in human blood erythrocytes was determined following the method of Quds et al. [5,32]. Cells from Section 2.3 were collected and lysed with ultrapure water at 4 °C. Absorbance readings at 630 nm (A_630_) and 700 nm (A_700_) were measured from 200 µL of the supernatant. Methemoglobin (MetHb) was prepared by mixing the same 200 µL of the sample solution with 10 µL of 5% potassium ferricyanide solution and the absorbance at 630 nm (A_100%metHb630_) and 700 nm (A_100%metHb700_) for MetHb was recorded. The oxidation rate of hemoglobin was then calculated using the following formula: metHb (%) = [(A_630_ − A_700_)/(A_100%metHb630_ − A_100%metHb700_)] × 100%.

### 2.7. Quantification of Protein Concentration, MDA Levels, LDH Activity, GSH/GSSG Ratio, and Activities of SOD, CAT, and GSH-Px in Human Erythrocytes

Following centrifugation of the erythrocyte reaction solution from Section 2.3 at 4000 rpm for 10 min, the supernatant was collected and stored at −80 °C. The cell sediment underwent three washes with PBS. Subsequently, complete cell lysis was achieved by adding 200 µL of ultrapure water at 4 °C, and the lysed cells were stored at −80 °C. Protein concentration, MDA levels, LDH activity, GSH/GSSG ratio, and activities of SOD, CAT, and GSH-Px in the lysed cells were determined using specific assay kits [5,33,34]. LDH enzyme activity in the supernatant was assessed separately using a kit, following the method described by Ma et al. [5,33].

### 2.8. Statistical Analysis

The data are expressed as mean ± SEM (standard error of the mean) and were derived from three to seven independent experiments. Statistical analysis was conducted using PRISM 6 statistical software (GraphPad Software, San Diego, CA, USA). One-way analysis of variance (ANOVA) followed by Dunnett’s test were utilized to compare the data with the vehicle control. A *p*-value of less than 0.05 was considered statistically significant.

## 3. Results and Discussion

### 3.1. Molecular Docking of Antioxidant Peptides with the ABTS Radical

**Figure 1:**
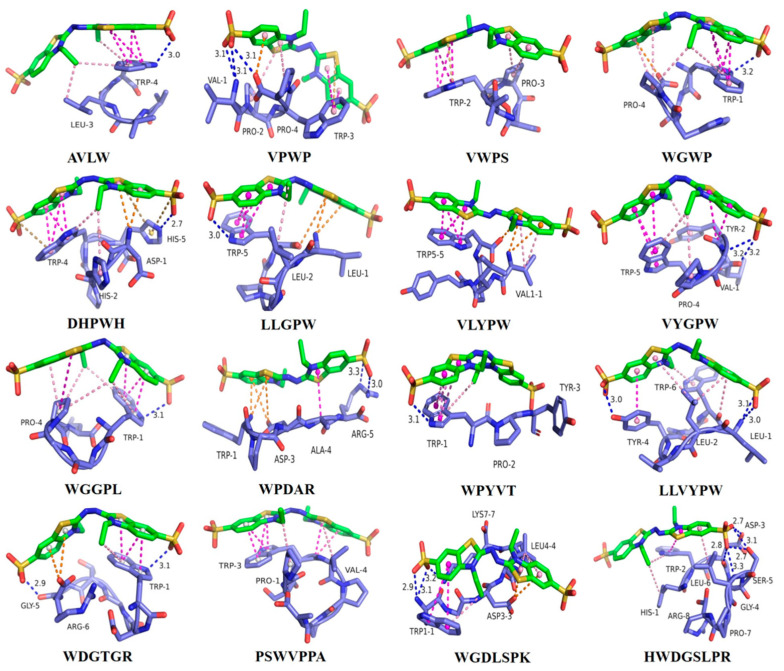
Optimal 3D model of docking between the sixteen ASE peptides and ABTS radical. The green compound is a free radical, and the purple compound is a proteolytic peptide. In the proteolytic peptide, the blue color represents nitrogen atoms, and the red color represents oxygen atoms. The dotted lines between the two compounds represent interaction forces: green indicates hydrogen bonding interactions, orange indicates electrostatic interactions, and red indicates hydrophobic interactions.

The molecular docking technique can be employed to investigate the structure–activity relationships, interaction sites, and mechanisms of peptides with free radicals [35,36]. Utilizing the minimum binding energy after docking as an index, the optimal binding configurations for the sixteen peptides were screened when docking with the ABTS radical, as depicted in Figure 1 (3D) and Figure S1 (2D). Table 1 elucidates the primary forces involved in the optimal conformation of these sixteen peptides upon docking with the ABTS radical, encompassing conventional hydrogen bonding, electrostatic interactions (Pi–Anion, Pi–Anion, Pi–Sulfur), and hydrophobic interactions (Pi–Pi stacking, Pi–Pi T-shape, Amide–Pi stacking, Pi–Alkyl, Pi–Sigma, Alkyl) [25,37]. Furthermore, the calculated minimum binding energies of the sixteen peptides were −3.2, −3.6, −3.7, −3.8, −4.1, −4.0, −4.3, −4.0, −3.8, −3.9, −3.7, −4.1, −4.2, −4.3, −4.7, and −4.1 kcal/mol upon docking with the ABTS radical, respectively. In a study by Wen et al. regarding active peptides from watermelon seeds, RDPEER, KELEEK, DAAGRLQE, LDDDGRL and GFAGDDAPRA exhibited notable chemical and cellular antioxidant activities with minimum binding energies of −3.87, −2.28, −2.73, −3.4, and −3.05 kcal/mol upon docking to the ABTS radical, respectively [3]. This finding indicates that all sixteen ASE antioxidant peptides can be tightly bound to the free radical and possess superior ABTS radical scavenging ability.

The key amino acid of the AVLW active site is Trp-4, which forms a hydrogen bond with the ABTS radical, as well as Pi–Pi stacked and Pi–Alkyl hydrophobic interactions. In the VPWP active site, the key amino acids are Val-1, Trp-3, and Pro-4, where the residues of Val-1 and Pro-4 form three hydrogen bonds, and the residues of Trp-3 and Pro-4 formed Pi–Sulfur and Pi–Anion electrostatic interactions. Similarly, in the VWPS active site, the key amino acids are Trp-2 and Pro-3, with Trp-2 residues forming Pi–Sulfur electrostatic interactions along with Pi–Pi stacked hydrophobic interactions. Moving to the WGWP active site, the key amino acid residues are Trp-1 and Pro-4, where Trp-1 residues form a hydrogen bond, Pi–Pi stacked, and Pi–Alkyl hydrophobic, interactions with the ABTS, while the residues of Pro-4 formed Pi–Anion electrostatic interactions along with Pi–Alkyl and Alkyl hydrophobic interactions with free radicals. In the DHPWH active site, the key amino acids are Trp-4 and His-5, where His-5 residues form a hydrogen bond and Pi–Sulfur electrostatic interactions with the ABTS radical, and Trp-4 residues form Pi–Sulfur electrostatic interactions, Pi–Pi stacked, and Pi–Alkyl hydrophobic interactions with the ABTS radical. At the LLGPW active site, the key amino acids are Leu-1 and Trp-5, where Trp-5 residues form a hydrogen bond, Pi–Sulfur electrostatic interaction, and Pi–Pi stacked hydrophobic interactions with the ABTS radical. The key amino acids in the VLYPW active site are Val-1 and Trp-5. The residues of these two amino acids form Pi–Cation and Pi–Anion electrostatic interactions with the ABTS radical, while the residues engage in Pi–Pi stacked and Pi–Alkyl hydrophobic interactions with the free radical. In the VYGPW active site, the key amino acids are Val-1, Tyr-2, Gly-3, and Trp-5. The residues of Val-1 and Gly-3 form two hydrogen bonds with the free radical, those of Tyr-2 engage in Amide–Pi stacked and Pi–Alkyl hydrophobic interactions, and those of Trp-5 form Pi–Pi stacked and Pi–Alkyl hydrophobic interactions with the ABTS radical. At the WGGPL active site, the key amino acids are Trp-1 and Pro-4, where Trp-1 residues form a hydrogen bond, Pi–Pi stacked, and Pi–Alkyl hydrophobic interactions with the ABTS radical. In the WPDAR active site, the key amino acids are Trp-1, Asp-3 and Arg-5. The residues of Arg-5 form two hydrogen bonds with the free radical, while those of Trp-1 and Asp-3 engage in Pi–Anion and Pi–Cation electrostatic interactions, respectively. At the WPYVT active site, the key amino acid is Trp-1, which forms a hydrogen bond, Pi–Pi stacked, and Pi–Alkyl hydrophobic interactions with the ABTS radical. In the LLVYPW active site, the key amino acids are Leu-1, Tyr-4 and Trp-6. Residues of Leu-1 and Tyr-4 form three hydrogen bonds with the free radical, while Trp-6 forms Pi–Anion electrostatic interactions with the ABTS radical. At the WDGTGR active site, the key amino acids are Trp-1, Gly-5 and Arg-6. Residues of Trp-1 and Gly-5 form two hydrogen bonds with the ABTS radical, and the residues of Arg-6 form Pi–Anion electrostatic interactions with the free radical. At the PSWVPPA active site, the key amino acid is Trp-3, which engages in Pi–Sulfur electrostatic interactions, Pi–Pi stacked, and Amide–Pi stacked hydrophobic interactions with the ABTS radical. At the WGDLSPK active site, the key amino acid is Trp-1 and Lys-7, which form three hydrogen bonds with the free radical. Finally, at the HWDGSLPR active site, the key amino acids are Trp-2, Asp-3, Ser-5, and Leu-6. Residues of Asp-3, Ser-5, and Leu-6 form four hydrogen bonds with the free radical, while residues of Trp-2 engage in Pi–Pi stacked and Pi–Alkyl hydrophobic interactions with the ABTS radical.

The docking results indicate that the hydrogen bonding interactions formed by tryptophan, proline, leucine, and valine with the ABTS radical, as well as the Pi–Pi stacked hydrophobic interactions, are crucial for the stability of the complex.

### 3.2. Molecular Docking of Antioxidant Peptides with the DPPH Radical

**Figure 2:**
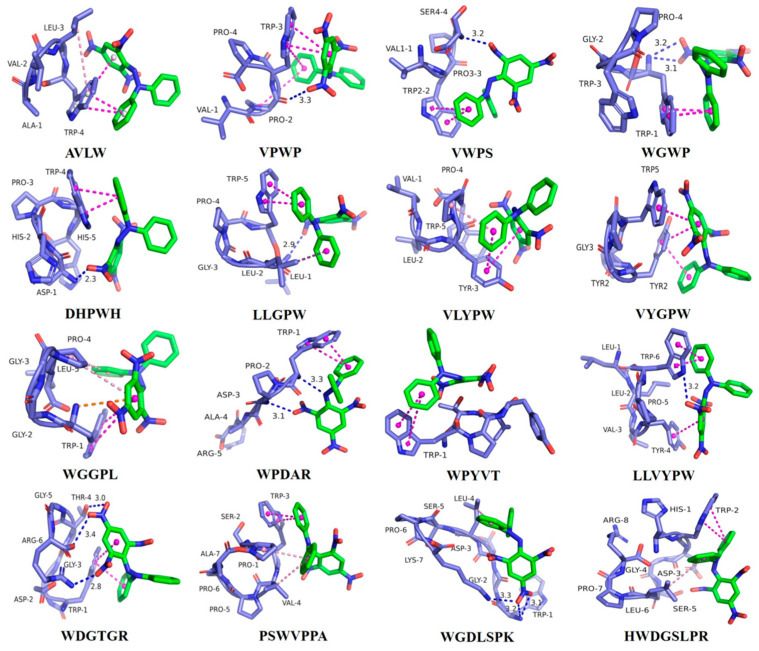
Optimal 3D model of docking between the sixteen ASE peptides and DPPH radical. The green compound is a free radical, and the purple compound is a proteolytic peptide. In the proteolytic peptide, the blue color represents nitrogen atoms, and the red color represents oxygen atoms. The dotted lines between the two compounds represent interaction forces: green indicates hydrogen bonding interactions, orange indicates electrostatic interactions, and red indicates hydrophobic interactions.

Using the minimum binding energy after docking as an index, the optimal binding conformations of sixteen peptides with the DPPH radical were screened, as illustrated in Figure 2 (3D) and Figure S2 (2D). The main forces involved in the optimal conformation of these sixteen peptides with the DPPH radical are demonstrated in Table 1, and encompass conventional hydrogen bonding, Pi–Cation electrostatic interaction, and hydrophobic interactions (Pi–Pi stacking, Pi–Pi T-shape, Amide–Pi stacking, Pi–Alkyl, and Pi–Sigma) [28]. Additionally, the minimum binding energies of these sixteen peptides after docking with DPPH molecules were determined to be −3.4, −3.3, −3.7, −3.8, −4.4, −4.0, −3.8, −3.7, −3.5, −3.7, −3.7, −4.3, −4.0, −4.2, −4.3, and −4.4 kcal/mol, respectively. It was noted by Wen et al., in their study of the active peptides in watermelon seeds, that RDPEER, KELEEK, DAAGRLQE, LDDDGRL, and GFAGDDAPRA exhibited enhanced chemical and cellular antioxidant activities, with minimum binding energies of −3.44, −1.99, −2.11, −3.32, and −2.79 kcal/mol for docking to the DPPH radical, respectively [3]. This finding suggests that all sixteen eel antioxidant peptides can tightly bind to the DPPH radical and possess strong DPPH radical scavenging abilities.

The key amino acid residue at the AVLW active site is Trp-4, which forms a hydrogen bond with the DPPH free radical, along with Pi–Pi stacked and Pi–Pi T-shaped hydrophobic interactions. At the VPWP active site, the critical amino acid is Trp-3, whose residue interacts with DPPH through a hydrogen bond, as well as Pi–Pi stacked and Pi–Pi T-shaped hydrophobic interactions. The VWPS active site features Trp-2 and Ser-4 as key amino acids, with the Ser-4 residue forming a hydrogen bond with DPPH, while Trp-2 engages in Pi–Pi stacked hydrophobic interactions with the free radical. The active site of WGWP is characterized by Trp-1, whose residue forms two hydrogen bonds and Pi–Pi stacked hydrophobic interactions with DPPH. DHPWH’s active site key amino acids are Asp-1 and Trp-4, with Asp-1 residue forming a hydrogen bond and Trp-4 residue engaging in Pi–Pi stacked hydrophobic interactions with DPPH. At the LLGPW active site, Leu-1 and Trp-5 are key amino acids, where Leu-1 residue forms a hydrogen bond with DPPH, and Trp-5 residue interacts with the free radical through Pi–Pi stacked hydrophobic interactions. The VLYPW active site features Tyr-3 and Pro-4 as key amino acids, whose residues form Pi–Pi T-shaped, Amide–Pi stacked, and Pi–Alkyl hydrophobic interactions with DPPH. At the VYGPW active site, Tyr-2 and Trp-5 are critical amino acids, where the Tyr-2 residue interacts with the free radical through Pi–Pi stacked and Pi–Pi T-shaped hydrophobic interactions, while the Trp-5 residue forms Pi–Pi stacked hydrophobic interactions. The key amino acid at the WGGPL active site is Trp-1, whose residue forms Pi–Cation electrostatic and Pi–Pi stacked hydrophobic interactions with the free radical. At the WPDAR active site, Trp-1 and Asp-3 are crucial amino acids, with their residues forming two hydrogen bonds and Pi–Pi stacked hydrophobic interactions with the free radical, respectively. The WPYVT active site’s key amino acid is Trp-1, whose residue interacts with the free radical through Pi–Pi stacked hydrophobic interactions. At the LLVYPW active site, Tyr-4 and Trp-6 are critical amino acids, where Trp-6 residue forms a hydrogen bond with the free radical, and Tyr-4 and Trp-6 residues engage in Pi–Pi stacked hydrophobic interactions with the free radical. The key amino acids at the WDGTGR active site are Trp-1, Thr-4, Gly-5, and Arg-6, with Thr-4, Gly-5, and Arg-6 residues forming three hydrogen bonds with the free radical, and the Trp-1 residue engaging in Pi–Pi stacked and Pi–Pi T-shaped hydrophobic interactions. PSWVPPA’s active site primarily consists of a Trp-3 residue, which interacts with the free radical through Pi–Pi stacked hydrophobic interactions. At the WGDLSPK active site, the critical amino acids are Trp-1 and Lys-7, whose residues form three hydrogen bonds with the free radical. Finally, the key amino acid at the HWDGSLPR active site is Trp-2, whose residue engages in Pi–Pi stacked and Pi–Pi T-shaped hydrophobic interactions with the free radical.

The docking results of sixteen peptides with ABTS and DPPH radicals were analyzed, revealing that hydrophobic amino acid residues, such as tryptophan, proline, leucine, and valine, serve as the primary binding sites for the peptides with the radicals. This observation underscores their significant contribution to the stability of the complexes and the modulation of the antioxidant properties of the peptides. The analysis of the active sites and interaction forces of these peptides with the free radical molecules highlights the crucial role of tryptophan in all sixteen peptides. Importantly, these findings align with the active sites anticipated by quantum chemistry calculations in earlier laboratory investigations (Figure S3).

### 3.3. Protective Effects of Sixteen ASE Antioxidant Peptides on the Morphological Characteristics of Human Erythrocytes

**Figure 3:**
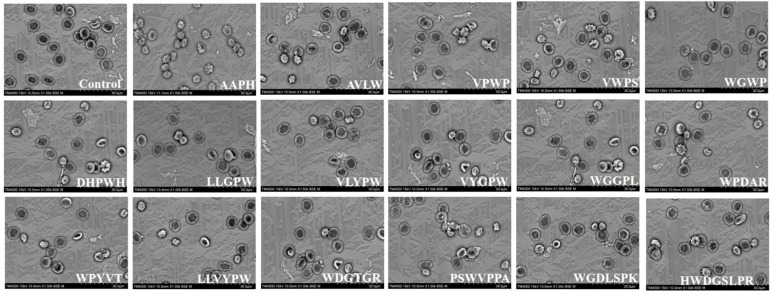
SEM micrographs of different treatment groups in the AAPH-induced erythrocyte hemolysis model.

To assess the protective effects of ASE antioxidant peptides against AAPH-induced morphological changes in human erythrocytes, the erythrocyte morphology was examined using scanning electron microscopy (SEM) [26,38]. It was observed that human erythrocytes in the control group exhibited a typical persimmon shape with a smooth surface and flat edges, forming a biconcave disc (Figure 3). Conversely, the cell membranes of the AAPH group experienced damage from the free radicals, leading to an imbalance of ions across the membrane [5]. This imbalance resulted in significant deformation, characterized by numerous spiny protrusions and rough surfaces. Additionally, some erythrocytes exhibited a rounded spherical shape, suggesting compromised fluidity of the phospholipid bilayer in the cell membrane, potentially leading to swelling and rupture, which are precursors to hemolysis [5]. In the group treated with ASE antioxidant peptides, the morphology of erythrocytes was partially preserved, with the majority maintaining a normal appearance. Only a few erythrocytes showed spiny protrusions or spherical shapes [5].

**Figure 4:**
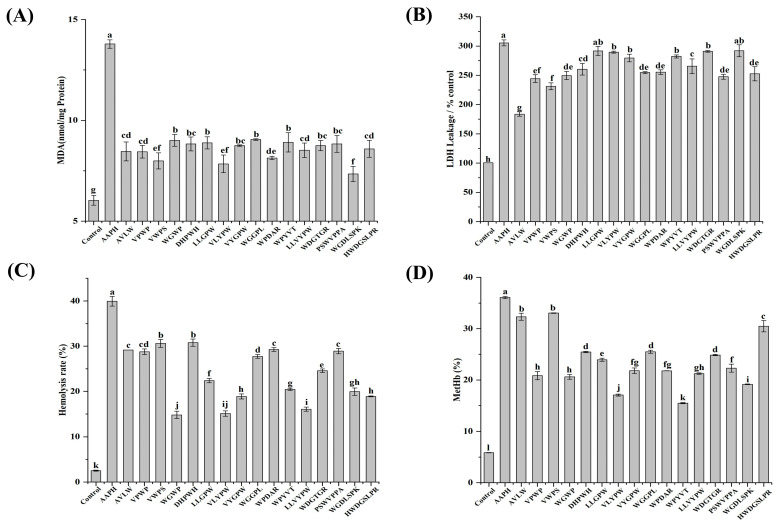

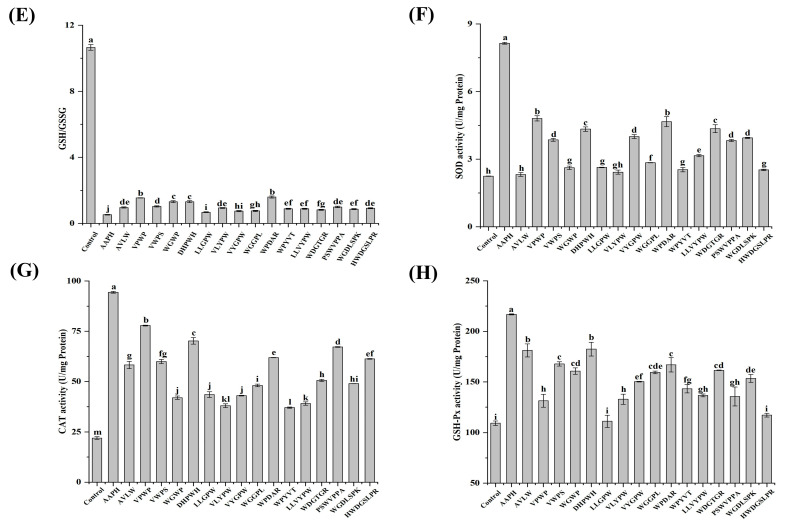
The protective effects and mechanisms of sixteen ASE peptides on AAPH-induced hemolysis of erythrocytes. Measurements include, as follows: MDA content (**A**); LDH leakage of the control group (**B**); erythrocyte hemolysis rate (**C**); MetHb percentage (**D**); GSH/GSSG ratio (**E**); SOD activity (**F**); CAT activity (**G**); and GSH-Px activity (**H**). Data points with different letters within the same test indicate significant differences (*p <* 0.05).

The integrity of cell membranes can be evaluated through the quantification of MDA production and LDH release [5,31]. The influence of ASE antioxidant peptides on the generation of MDA and the release of LDH is illustrated in Figure 4A,B. In the control group, MDA levels were quantified at 6.03 ± 0.24 nmol/mg protein, whereas in the AAPH group, MDA production showed a significant increase to 13.93 ± 0.20 nmol/mg protein, accompanied by LDH release that reached 305.45 ± 5.03% of the control group. The elevated MDA and LDH levels indicate significant cellular damage relative to the control group. Conversely, all peptide-treated groups showed a marked decrease in MDA and LDH levels when compared to the AAPH group (*p <* 0.05). The protective effect was most pronounced with peptide WGDLSPK, yielding an MDA production of 7.34 ± 0.38 nmol/mg of protein, followed by peptides VLYPW and VWPS, with MDA productions of 7.84 ± 0.44 nmol/mg of protein and 7.99 ± 0.40 nmol/mg of protein, respectively. Peptides AVLW (183.73 ± 3.70% of the control group), VWPS (231.20 ± 5.91% of the control group), and VPWP (244.31 ± 6.70% of the control group) displayed reduced LDH leakage, which can potentially be attributed to their smaller molecular weights.

In Figure 4C,D, it is evident that the hemolysis and hemoglobin oxidation rates were markedly lower in the control group, with rates recorded at only 2.52 ± 0.11% and 5.85 ± 0.03%, respectively. Conversely, the hemolysis rate (39.95 ± 1.08%) and hemoglobin oxidation rate (36.09 ± 0.23%) of erythrocytes were substantially higher in the AAPH group. Notably, in all peptide-treated groups, the hemolysis and hemoglobin oxidation rates were significantly lower compared to the AAPH-induced injury group, indicative of the peptides’ efficacy in safeguarding erythrocytes from oxidative damage (*p <* 0.05). Among the peptide variants, erythrocytes treated with VLYPW showed a significantly lower hemolysis rate (15.11 ± 0.26%) and hemoglobin oxidation rate (17.06 ± 0.16%), highlighting its strong protection against oxidative hemolysis. Moreover, WGWP, VYGPW, LLVYPW, and WGDLSPK also demonstrated superior efficacy in inhibiting the hemolysis rate and hemoglobin oxidation. Additionally, the ABTS free radical uptake capacity in different antioxidant peptide treatment groups exhibited a negative correlation with the hemolysis rate and MetHb level, while the ORAC value displayed a negative correlation with the MetHb level (Tables S1 and S2). This underscores the potential of ASE-derived antioxidant peptides in mitigating erythrocyte hemolysis and hemoglobin oxidation through their antioxidative properties.

### 3.4. Effect of Sixteen ASE Antioxidant Peptides on Erythrocyte Redox Systems (GSH/GSSG, SOD, CAT, GSH-Px) under AAPH-Induced Oxidative Stress

GSH typically functions as the primary defense mechanism against oxidative stress, with its levels commonly utilized as indicators in assessing cellular oxidative stress [31,39]. As illustrated in Figure 4E, a notably higher GSH/GSSG ratio of 10.66 ± 0.17% was observed in the control group, indicating that elevated GSH levels are naturally maintained by erythrocytes under normal physiological conditions. In contrast, a substantial decrease in the GSH/GSSG ratio to 0.53 ± 0.02% was observed in the AAPH group. Nevertheless, the GSH/GSSG ratios were markedly higher in all peptide-treated groups compared to the AAPH group (*p <* 0.05), suggesting that cellular oxidative damage was effectively mitigated by these peptides through the enhancement of the GSH/GSSG ratio. Remarkably, the WPDAR- and VPWP-treated groups exhibited the highest GSH/GSSG ratios of 1.60 ± 0.06% and 1.54 ± 0.01%, respectively, significantly surpassing those of the other peptide-treated groups, followed by the WGWP- and DHPWH-treated groups, both demonstrating GSH/GSSG ratios of 1.31 ± 0.06%.

Erythrocytes are rich in enzymatic antioxidant systems (SOD, CAT, GSH-Px, etc.) that protect against oxidative damage [40,41]. As demonstrated in Figure 4F–H, intracellular SOD, CAT, and GSH-Px activities exhibited a substantial increase in the AAPH-treated group (*p* < 0.05), being 3.62-fold, 4.30-fold, and 1.99-fold higher than those in the control group, respectively. This increase was due to the activation of the enzymatic defense system in erythrocytes triggered by AAPH, resulting in a higher demand for antioxidant enzymes. The peptide-treated groups showed different reductions in enzyme activities compared to the AAPH-treated group. The VLYPW-treated group exhibited diminished SOD (2.43 ± 0.09 U/mg protein), CAT (37.98 ± 0.93 U/mg protein), and GSH-Px (132.85 ± 5.07 U/mg protein) activities. Similarly, LLGPW, WPYVT, LLVYPW, and HWDGSLPR also manifested reduced antioxidant enzyme activities in the peptide-treated group, which can possibly be attributed to the potent free radical scavenging ability of the ASE antioxidant peptides, effectively attenuating the demand for antioxidant enzymes during oxidative stress.

### 3.5. Mechanistic Study on the Inhibition of AAPH-Induced Oxidative Damage in Erythrocytes by ASE Antioxidant Peptides

**Figure 5:**
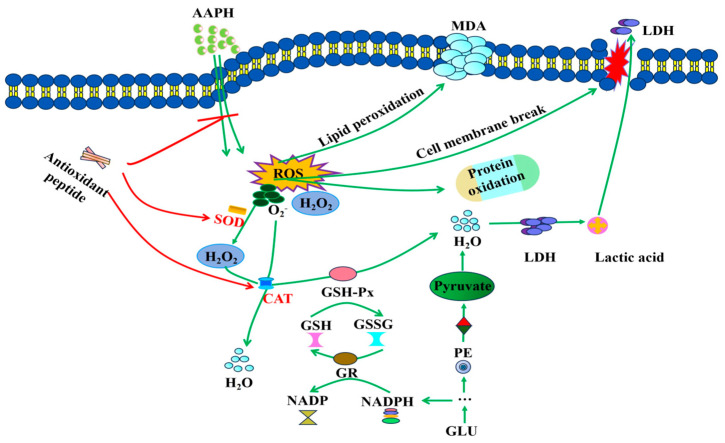
Possible intracellular antioxidant mechanisms of sixteen ASE peptides in mitigating AAPH-induced oxidative damage. The green markers indicate AAPH-induced oxidative damage, while the red markers illustrate the mechanism of action of antioxidant peptides.

As shown in Figure 5, the antioxidant mechanism by which ASE peptides inhibit AAPH-induced oxidative damage in human erythrocytes was hypothesized based on changes in various indicators in human blood erythrocytes [5,42,43].

AAPH functions as a free radical initiator, triggering the generation of substantial ROS upon interaction with erythrocytes, including O_2_^−^ and H_2_O_2_. Excessive ROS interact with unsaturated fatty acids in the cell membrane, leading to significant MDA production. MDA then cross-links with membrane phospholipids and proteins, causing morphological changes in erythrocytes [5,33]. This disruption of the phospholipid bilayer impairs the cell membrane, leading to the release of LDH and hemoglobin into the extracellular space, ultimately causing hemolysis [5,30]. Antioxidant peptides directly react with peroxyl radicals produced by AAPH and reduce their levels in hemoglobin [5]. GSH plays a crucial role as a non-enzymatic antioxidant in erythrocytes, serving as the primary defense against oxidative stress. In the presence of GSH-Px, GSH undergoes oxidation to form GSSG. Excessive GSSG can be converted back to GSH with the help of NADPH and glutathione reductase (GR), effectively inhibiting lipid peroxidation [5,31]. Additionally, erythrocytes are equipped with enzymatic antioxidant defense systems such as SOD, CAT, and GSH-Px. SOD converts O_2_^−^ to H_2_O_2_, which is then further reduced to water by CAT. GSH-Px converts H_2_O_2_ to water or reacts with GSH to generate water [5,31]. Non-enzymatic and enzymatic reactions maintain a dynamic balance between intracellular free radical generation and scavenging, directly or indirectly participating in redox reactions to preserve the normal physiological functions of erythrocytes. However, the extended exposure of erythrocytes to free radicals surpasses the capacity of the intracellular defense system to scavenge excess free radicals, leading to erythrocyte destruction. The inclusion of ASE antioxidant peptides effectively reduces the oxidative damage caused by free radicals in erythrocytes, restoring the balance between enzymatic and non-enzymatic antioxidants and thus inhibiting AAPH-induced oxidative damage. This suggests that ASE antioxidant peptides have the potential to ameliorate sickle cell anemia and reduce erythrocyte storage damage [22,23,24].

Among the sixteen ASE antioxidant peptides studied, VLYPW demonstrated exceptional chemo-antioxidant activity and protected human blood erythrocytes morphologically. This effect is attributed to its higher levels of tyrosine and tryptophan content, along with an increased proportion of hydrophobic amino acids [5,44]. This elevated concentration of hydrophobic amino acids might enhance cellular permeability, thereby boosting intracellular antioxidant activity [4,5].

## 4. Conclusions

Molecular docking of the sixteen peptides with ABTS and DPPH free radicals revealed that hydrophobic amino acids within the peptides, such as tryptophan, proline, leucine, and valine residues, play a crucial role in the stability of the complexes. These residues may be important factors influencing the chemical antioxidant properties of the peptides. Investigations using a human erythrocyte oxidative damage model indicated that the sixteen peptides can provide protective effects against oxidative damage in human erythrocyte by reacting directly with peroxyl radicals and modulating both non-enzymatic and enzymatic antioxidant systems. These findings elucidate the chemical and cell-based antioxidant mechanisms of the peptides, offering a theoretical basis for studying their functional activity and for the development of high value-added products.

## Figures and Tables

**Table 1:** Docking sites and interaction forces between the ASE antioxidant peptides and radical receptor.

	Peptide	CDOCKER Energy (kcal/mol)	Hydrogen Bond	Electrostatic Interaction			Hydrophobic Interaction					
			Conventional Hydrogen Bond	Pi–Anion	Pi–Sulfur	Pi–Cation	Pi–Pi Stacked	Pi–Pi T-Shaped	Amide–Pi Stacked	Pi–Alkyl	Pi–Sigma	Alkyl
**ABTS**	AVLW	−3.2	W4				W4-2			W4-2		L3
	VPWP	−3.6	V1-2 *, P4	P4	W3			W3-2		P2-2, P4		
	VWPS	−3.7			W2		W2-4			P3-2		
	WGWP	−3.8	W1	P4			W1-4			W1, P4-2		P4
	DHPWH	−4.1	H5		W4, H5-2	D1-2	W4-4			H2, W4		
	LLGPW	−4.0	W5	L1-2	W5		W5-4					L2
	VLYPW	−4.3		W5-1		V1-2	W5-4			V1-2		
	VYGPW	−4.0	V1, G3				W5-3		Y2-2	Y2, W5		P4
	WGGPL	−3.8	W1				W1-3			W1-2, P4	P4	P4-2
	WPDAR	−3.9	R5-2	W1-2		D3-2			A4			
	WPYVT	−4.1	W1				W1-4			W1		
	LLVYPW	−4.2	L1-2, Y4	W6			Y4			L2-4		
	WDGTGR	−4.7	W1, G5	R6-2			W1-3			W1		
	PSWVPPA	−4.1			W3		W3-4		W3-2	V4		P1
	WGDLSPK	−3.9	W1-2, K7	D3-2			W1-2			W1, L4-2		
	HWDGSLPR	−4.4	D3-2, S5-2, L6				W2			H1, W2, L6		
**DPPH**	AVLW	−3.4					W4-2	W4-1		L3		
	VPWP	−3.3	W3				W3-2	W3	P2			
	VWPS	−3.7	S4				W2-2					
	WGWP	−3.8	W1-2				W1-2					
	DHPWH	−4.4	D1				W4-2					
	LLGPW	−4.0	L1				W5-2				L2	
	VLYPW	−3.8						W3	W3	P4		
	VYGPW	−3.7					Y2, W5	Y2				
	WGGPL	−3.5				W1	W1-2			P4-2		
	WPDAR	−3.7	W1, D3				W1-2					
	WPYVT	−3.7					W1-2					
	LLVYPW	−4.3	W6				Y4, W6-2					
	WDGTGR	−4.0	T4, G5, R6				W1	W1				
	PSWVPPA	−4.2					W3-2			P1, V4		
	WGDLSPK	−4.3	W1-2, K7							L4		
	HWDGSLPR	−4.4					W2-2	W2		L6		

* V1 indicates that valine is the first amino acid of the peptide, while 2 indicates the formation of two hydrogen bonds with free radicals.

## Data Availability

The data are contained within the article and Supplementary Materials. Other original data supporting the reported results are available upon request.

## Supplementary Materials

The following supporting information can be downloaded at: https://www.mdpi.com/article/10.3390/antiox13080888/s1, Figure S1: Optimal 2D model of docking between sixteen ASE peptides and ABTS radical; Figure S2: Optimal 2D model of docking between sixteen ASE peptides and DPPH radical; Figure S3: Active-site analysis of the sixteen ASE peptides by HOMO and Mulliken charge distribution, and bond length. Table S1: Antioxidant activities of the sixteen ASE peptides. Significant differences between groups are denoted by different letters within the same test *(p* < 0.05).; Table S2: Correlation study of chemical antioxidant activity with various indices in erythrocyte models. * Significant correlations at *p <* 0.05. ** Significant correlations at *p* < 0.01.
