# Supplementary material for: Investigation of Antioxidant Mechanisms of Novel Peptides Derived from Asian Swamp Eel Hydrolysate in Chemical Systems and AAPH-Induced Human Erythrocytes
Source: Antioxidants (Basel). 2024 Jul 23;13(8):888. doi: 10.3390/antiox13080888 (PMC11351846; doi:10.3390/antiox13080888)
Supplement: Supplementary file 1 [file antioxidants-13-00888-s001.zip › antioxidants-3102182-supplementary.pdf]

Supplementary Materials

Investigation of Antioxidant Mechanisms of Novel Peptides Derived from Asian Swamp Eel Hydrolysate in Chemical Systems and AAPH-Induced Human Erythrocytes

Xiao Wang [1,†], Bingjie Chen [1,†], Khushwant S. Bhullar [2], Hang Yang [3], Xiaohu Luo [4], Juan Fu [1], Hongru Liu [1], Di Su [5], Dapeng Sun [1], Yongjin Qiao [1,*] and Wenzong Zhou [3,*]

[1] Crop Breeding and Cultivation Research Institution, Research Center for Agricultural Products Preservation and Processing, Shanghai Academy of Agricultural Sciences, Shanghai 201403, China; zhenhans2005@gmail.com (X.W.); chenbingjie0204@126.com (B.C.); maryfly@live.cn (J.F.); 20200203@saas.sh.cn (H.L.); sundapeng@saas.sh.cn (D.S.)

[2] Department of Agricultural Food & Nutritional Science, University of Alberta, Edmonton, AB T6G 2P5, Canada; bhullar@ualberta.ca

[3] Key Laboratory of Integrated Rice-Fish Farming Ecosystem, Ministry of Agriculture and Rural Affairs, Shanghai Academy of Agricultural Sciences, Shanghai 201403, China; yanghangqu@saas.sh.cn

[4] Zhejiang-Malaysia Joint Research Laboratory for Agricultural Product Processing and Nutrition, College of Food Science and Engineering, Ningbo University, Ningbo 315832, China; xh06326@gmail.com

[5] School of Pharmacy, Shanghai Jiao Tong University, 800 Dongchuan Road, Shanghai 200240, China; sudi@sjtu.edu.cn

* Correspondence: qiaoyongjin@hotmail.com (Y.Q.); wzzhou505@sina.com (W.Z.); Tel: +86-21-6220-1847 (Y.Q.); +86-21-6220-2634 (W.Z.)

† These authors contributed equally to this work.

Table S1. Antioxidant activities of the sixteen ASE peptides.

Peptide Sequence	ABTS Assay (μmol TE/μmol)	ORAC Assay (μmol TE/μmol)
AVLW	1.5164 ± 0.0430 [h]	3.2718 ± 0.2078 [d]
VPWP	1.3751 ± 0.0122 [i]	2.3808 ± 0.0059 [e]
VWPS	0.9421 ± 0.0153 [k]	1.9430 ± 0.0417 [f]
WGWP	2.2033 ± 0.0611 [e]	4.3527 ± 0.2014 [c]
DHPWH	1.1705 ± 0.0100 [j]	2.0345 ± 0.0854 [f]
LLGPW	1.2663 ± 0.0395 [ij]	2.3873 ± 0.0617 [e]
VLYPW	3.1599 ± 0.0295 [b]	5.0020 ± 0.0039 [b]
VYGPW	2.6790 ± 0.1061 [d]	5.4949 ± 0.1416 [a]
WGGPL	1.2424 ± 0.0374 [j]	3.0565 ± 0.0120 [d]
WPDAR	1.5669 ± 0.0243 [gh]	3.1814 ± 0.0701 [d]
WPYVT	3.0420 ± 0.1698 [c]	5.6709 ± 0.1225 [a]
LLVYPW	3.2836 ± 0.1267 [a]	4.1374 ± 0.1811 [c]
WDGTGR	1.5951 ± 0.0016 [gh]	3.1841 ± 0.0410 [d]
PSWVPPA	1.5085 ± 0.0483 [h]	2.4024 ± 0.1206 [e]
WGDLSPK	1.6648 ± 0.0552 [fg]	3.0847 ± 0.0021 [d]
HWDGSLPR	1.7647 ± 0.0205 [f]	1.9581 ± 0.1244 [f]

Data with different letters in the same test indicate significant differences ($p < 0.05$).

Table S2. Correlation study of chemical antioxidant activity with various indices in erythrocyte models.

	ABTS Assay	ORAC Assay
MDA content	0.249	0.381
LDH Leakage of control	0.574*	0.604*
Hemolysis rate	−0.691**	−0.476
MetHb (%)	−0.653**	−0.625**
GSH/GSSG	−0.312	−0.383
SOD activity	−0.296	−0.299
CAT activity	−0.615*	−0.617*
GSH-Px activity	−0.363	−0.177

* Significant correlations at $p < 0.05$. ** Significant correlations at $p < 0.01$.

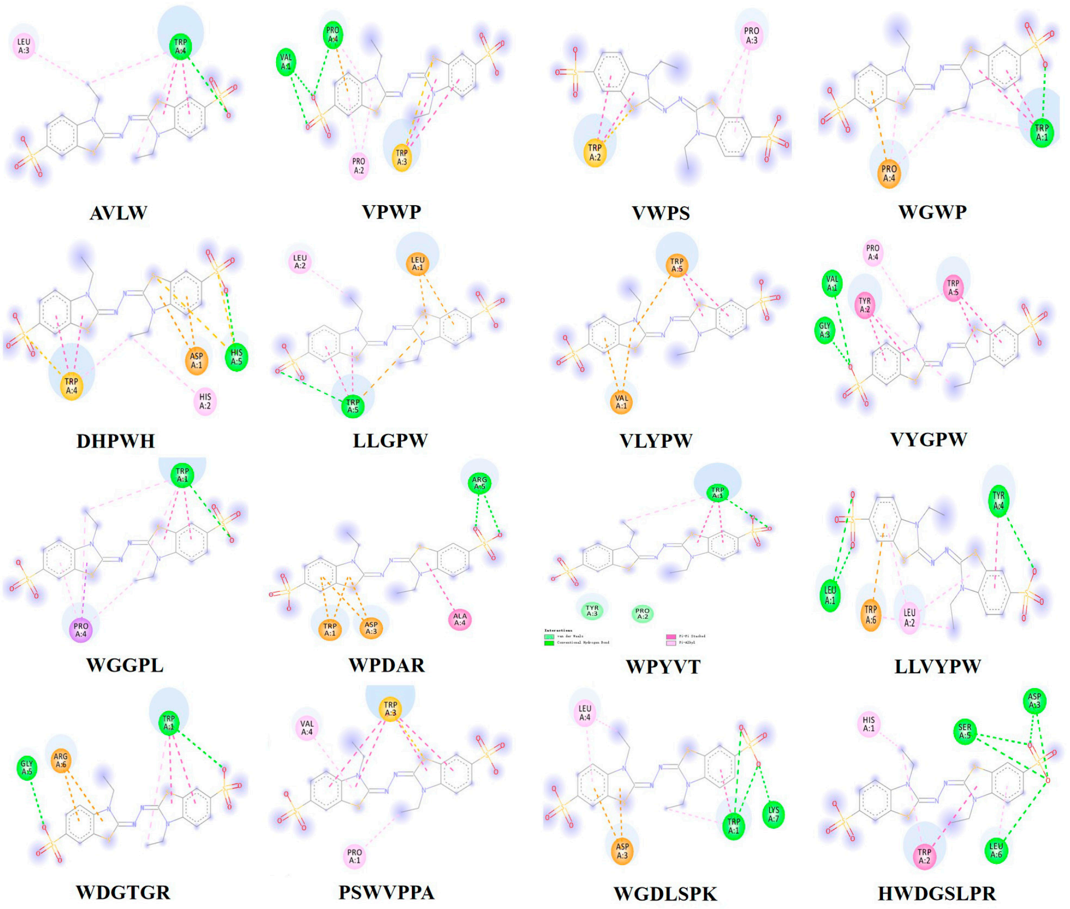

Figure S1. Optimal 2D model of docking between sixteen ASE peptides and ABTS radical.

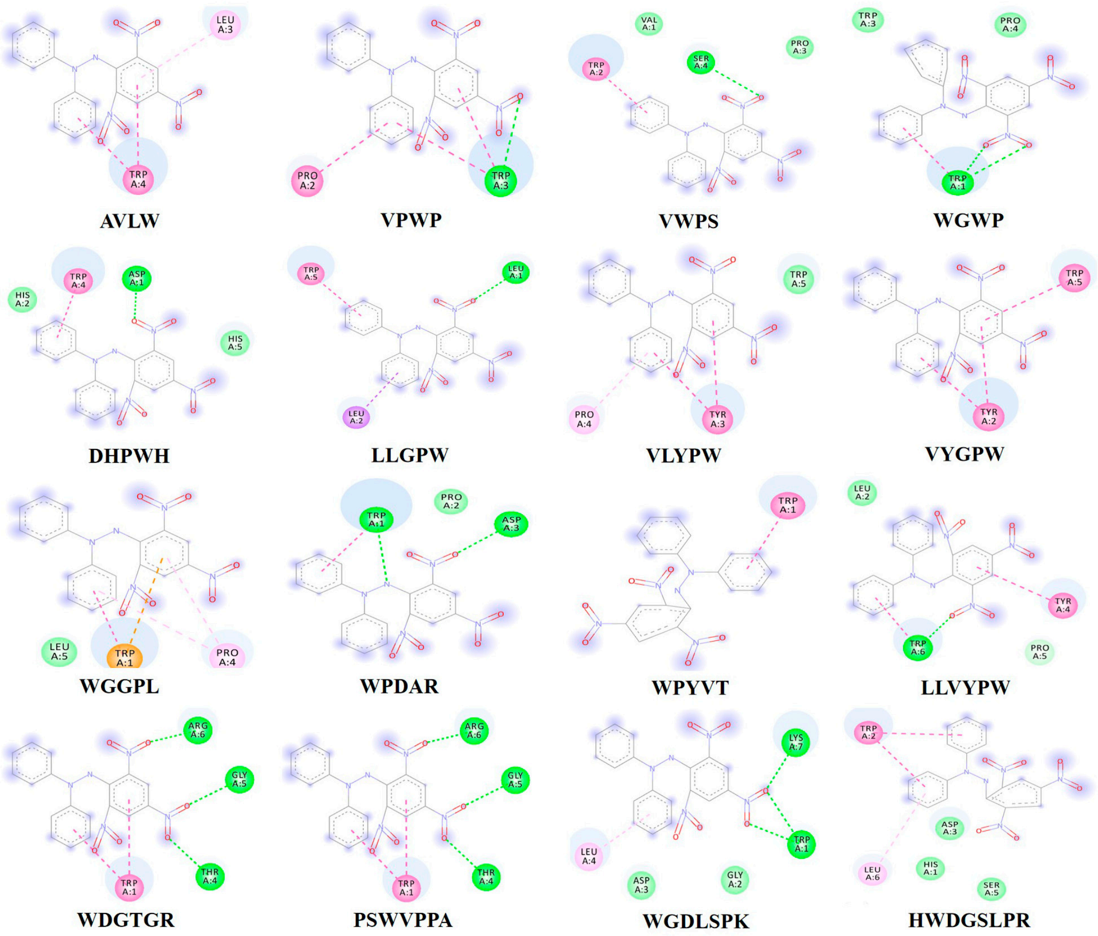

Figure S2. Optimal 2D model of docking between sixteen ASE peptides and DPPH radical.

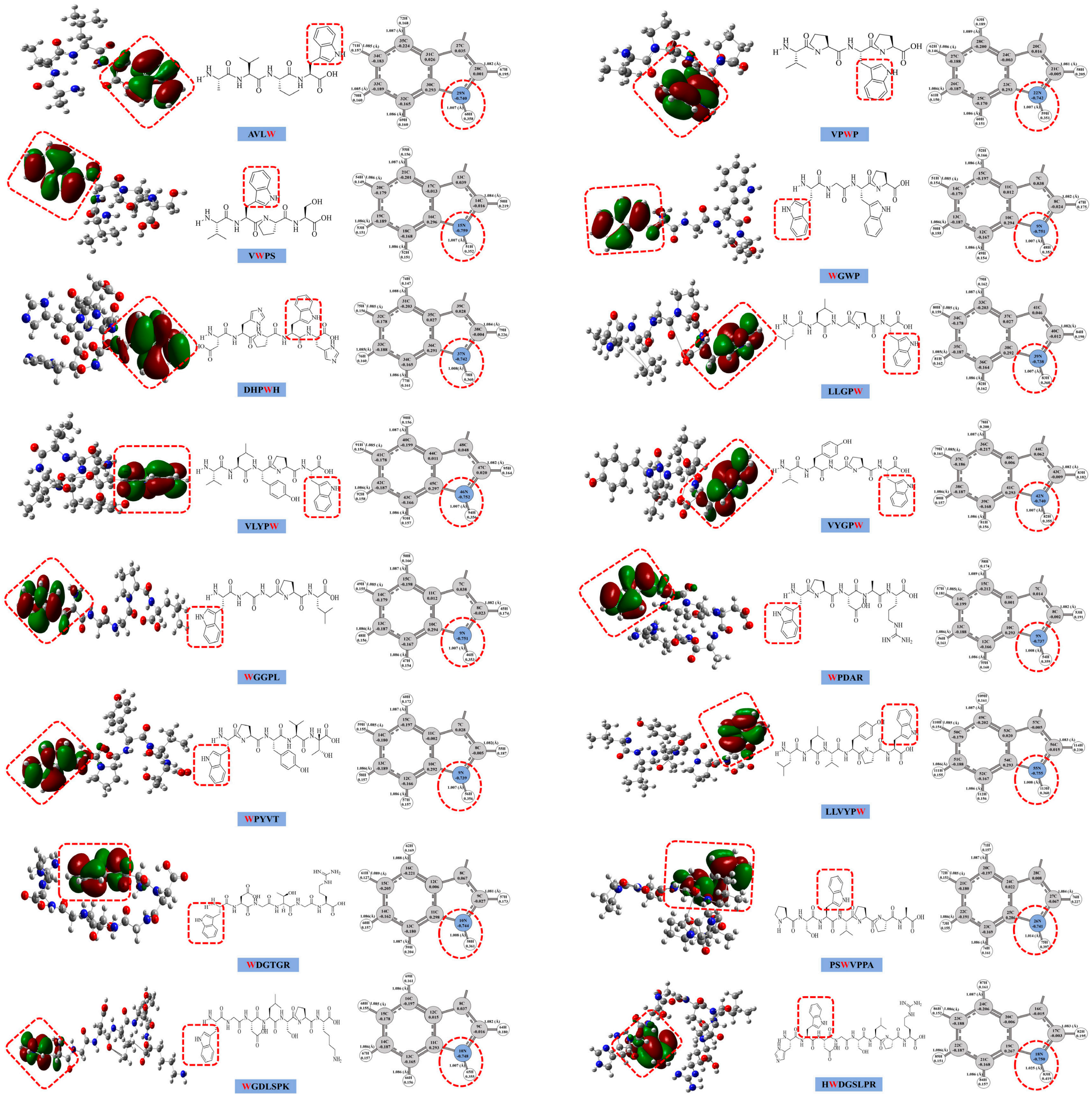

Figure S3. Active-site analysis of the sixteen ASE peptides by HOMO and Mulliken charge distribution, and bond length.
